# *Paraburkholderia phytofirmans* PsJN triggers local and systemic transcriptional reprogramming in *Arabidopsis thaliana* and increases resistance against *Botrytis cinerea*

**DOI:** 10.3389/fpls.2025.1554036

**Published:** 2025-06-03

**Authors:** Sofija Nešić, Dragana Bosnić, Jelena Samardžić, Ivana Nikolić, Aziz Aziz, Dragana Nikolić

**Affiliations:** ^1^ Group for Plant Molecular Biology, Institute of Molecular Genetics and Genetic Engineering, Department of Microbiology and Plant Biology, University of Belgrade, Belgrade, Serbia; ^2^ Induced Resistance and Plant Bioprotection (RIBP), University of Reims Champagne Ardenne, UFR Sciences, Reims, France

**Keywords:** *Arabidopsis thaliana*, beneficial bacteria, *Botrytis cinerea*, roots, systemic response, transcriptome analysis, miRNA, priming

## Abstract

Fungal pathogens are one of the main causes of yield losses in many crops, severely affecting agricultural production worldwide. Among the various approaches to alleviate this problem, beneficial microorganisms emerge as an environmentally friendly and sustainable alternative. In addition to direct biocontrol action against pathogens, certain plant growth-promoting bacteria (PGPB) enhance the plant immune defense to control diseases through induced systemic resistance (ISR). *Paraburkholderia phytofirmans* PsJN has been shown as an efficient biocontrol agent against diseases. However, the specific mechanisms underlying these beneficial effects at both local and systemic level remain largely unknown. In this study, we investigated the transcriptional response of *Arabidopsis thaliana* at above- and below-ground levels upon interaction with *P. phytofirmans* PsJN, and after *Botrytis cinerea* infection. Our data clearly support the protective effect of *P. phytofirmans* PsJN through ISR against *B. cinerea* in plants grown in both soil and hydroponic conditions. The comparative transcriptome analysis of the mRNA and miRNA sequences revealed that PsJN modulates the expression of genes involved in abiotic stress responses, microbe-plant interactions and ISR, with ethylene signaling pathway genes standing out. In roots, PsJN predominantly downregulated the expression of genes related to microbe perception, signaling and immune response, indicating that PsJN locally provoked attenuation of defense responses to facilitate and support colonization and the maintenance of mutualistic relationship. In leaves, the increased expression of defense-related genes prior to infection in combination with the protective effect of PsJN observed in later stages of infection suggests that bacterial inoculation primes plants for enhanced systemic immune response after subsequent pathogen attack.

## 1 Introduction

Harnessing plant-associated microbes and their role in plant health represents a promising environment friendly strategy for a sustainable agriculture. Most of beneficial bacteria including the plant growth promoting bacteria (PGPB) associated with plants in the close vicinity of their roots (rhizosphere) or within the plant tissues (as endophytes) play a crucial role in plant health and productivity. These bacteria promote plant growth and prime plants for enhanced resistance against abiotic and biotic stress (Lugtenberg and Kamilova, 2009). The beneficial bacteria-mediated resistance relies mainly on the priming process of plant immune system (Pieterse et al., 2014; Conrath et al., 2015; Gruau et al., 2015). However, little headway has been made in identifying the molecular mechanisms involved in the primed mechanism linked to PGPB-induced resistance.

Plant immunity is a complex system that is a result of a co-evolution between plants and pathogens. It has been shown that interaction with beneficial bacteria can help plants to better react to a subsequent pathogen attack (Pieterse et al., 2014). This defense strategy triggered by PGPB, to prime plants against pathogens, is called induced systemic resistance (ISR). As a result, plants develop an earlier and stronger defense reaction to the pathogen attack, delaying symptom development, reducing disease severity and inhibiting pathogen growth (van Loon, 2007; Cartieaux et al., 2008).

Phytopathogenic fungi are a dominant cause of plant diseases. *Botrytis cinerea* is a phytopathogenic fungus affecting a wide range of hosts. As an aggressive necrotroph, it is responsible for major crop loss every year (Bi et al., 2023). Preventing this pathogen, particularly at an early stage, is of great importance. PGPB like *Bacillus cereus* AR156, *Burkholderia* sp. AU4i, *Pseudomonas fluorescens* PTA-CT2 and *Paraburkholderia phytofirmans* PsJN have been shown to increase plant tolerance to this pathogen in Arabidopsis and grapevine (Miotto-Vilanova et al., 2016; Nie et al., 2017; Nguyen et al., 2020; Colavolpe et al., 2021).

*P. phytofirmans* PsJN (previously known as *Burkholderia phytofirmans* PsJN) has been shown to colonize a wide variety of plant species including grapevine, rice, and tomato, as well as *Arabidopsis thaliana* (Poupin et al., 2013; Issa et al., 2018; Timmermann et al., 2019; King et al., 2023). *P. phytofirmans* PsJN also activates plant defense system in different crop species and in *A. thaliana*, thereby reducing their susceptibility to various pathogens (Timmermann et al., 2017; Colavolpe et al., 2021). This beneficial bacterial strain dwells in the rhizosphere as well as within plant tissues as an endophyte, supporting its beneficial affect in promoting plant growth and their resistance to various abiotic and biotic stresses. Poupin et al. (2013) have shown that a single inoculation of Arabidopsis with PsJN influences the whole life cycle of the plant, accelerating growth rate and shortening vegetative period, both of which are important for crop production. PsJN enhances plant growth and health through various mechanisms, including the production of growth-promoting phytohormones like auxins, the enzyme 1-aminocyclopropane-1-carboxylic acid (ACC) deaminase that reduces stress hormone ethylene levels, and the secretion of siderophores in the rhizosphere that increase iron availability (Esmaeel et al., 2018).

Exploring the molecular mechanisms of plant-microbiota interactions is essential for understanding how plants respond to beneficial microbes. Global transcriptome analyses on PsJN effects in plants are scarce, leaving a gap in understanding the mechanisms involved in beneficial effect of PsJN. In the study investigating PsJN-Arabidopsis interaction, before pathogen challenge, 405 genes (corresponding to 1.8% of the analyzed Arabidopsis genome) were regulated by the bacterium. A principal component analysis showed that PsJN-induced plant responses to the pathogen could be differentiated from those induced by the pathogen itself. A network comprising four gene clusters was changed by PsJN, including genes related to jasmonate, ethylene, salicylic acid and ROS pathways (Timmermann et al., 2019). Another research highlights the distinct effects of PsJN and native *Burkholderia* endophytes found in rice (King et al., 2023). The colonization of the rice endosphere by PsJN elicits a unique transcriptomic response compared to native strains. This response encompasses alterations in genes associated with secondary metabolism, immunity, and phytohormones (King et al., 2023). The molecular and biochemical responses of *A. thaliana* plants inoculated with the PsJN strain were examined under both short-term and long-term salt stress throughout their entire life cycle. The findings revealed transcriptional responses linked to salt tolerance, including a rapid activation of general abiotic stress-responsive genes, and the transcriptional regulation of genes related to nutrient uptake (Pinedo et al., 2015). Future research should explore whether these insights can be applied to other plant models and beneficial microbes, thereby enhancing the potential of microbiome-based strategies for improving crop production and resilience.

One of the key regulators of plant development, growth and stress response are small RNAs (Borges and Martienssen, 2015; Waheed et al., 2021). They are known to modulate their target genes, adjusting their expression, and thus the response of plants to their environment (Axtell and Bowman, 2008). Micro RNAs are a class of small non-coding RNAs that regulate expression of the target genes through induced mRNA degradation and translational repression (Iwakawa and Tomari, 2013; Li et al., 2013b; Middleton et al., 2021). sRNAs have been shown to play a positive role in plant immune defense against various pathogenic microbes, including bacteria, fungi and oomycetes (Kong et al., 2022).

In this study we evaluated the *P. phytofirmans* PsJN activity in enhancing both *A. thaliana* resistance to the necrotrophic fungus *B. cinerea* and plant growth, and explored mechanisms underlying PsJN-mediated resistance by analyzing transcriptional alterations occurring in *A. thaliana* after challenge with *B. cinerea*. We more especially focused on transcriptional response using mRNA and sRNA sequencing in both roots and leaves upon interactions with *P. phytofirmans* PsJN and after pathogen challenge.

## 2 Materials and methods

### 2.1 Plant material and growth conditions

Seeds of *Arabidopsis thaliana* Col-0 were surface-sterilized with 70% (v/v) ethanol for 5 min, followed by 20 min incubation in a sterilizing solution (10% commercial bleach, 0.05% (v/v) Tween 20, dH_2_O) (Nikolić et al., 2023). The seeds were washed 3 times with sterile distilled water (dH_2_O). After 5 days of stratification in the dark at +4°C, seeds were placed on soil and covered with cling foil for two weeks for optimal humidity during germination. Plants were grown under short-day regime (10h day/ 14h night), light intensity of 150 μmol m^−2^s^−1^, relative humidity of 55-65%, day/night temperatures of 21°C/19°C. Arabidopsis plants were also grown hydroponically following protocol Conn et al. (2013). In hydroponics, plants were grown under the same conditions (light intensity, day length and humidity) as plants grown in soil.

### 2.2 Bacterial growth conditions and plant inoculation

*Paraburkholderia phytofirmans* PsJN (obtained from Leibniz Institute DSMZ - German Collection of Microorganisms and Cell Cultures) was grown in LB or minimal media (Coleman et al., 2002) for 18 h, in an orbital shaker (150 rpm) at 30°C. For soil drench inoculation, bacteria were pelleted at 2500 g/4°C for 15 min, washed and resuspended in 10 mM MgSO_4_. Three-week-old plants were soil-drenched with PsJN to reach a final concentration of 1 × 10^8^ CFU/g soil. The same volume of 10 mM MgSO_4_ was used as a mock treatment (control). For hydroponic inoculation, bacteria were grown in minimal media pelleted at 1500 g/4°C for 15 min and washed with hydroponic medium. PsJN suspension was added to the germination medium during the first week of seedling growth. Adult plants were inoculated two weeks prior to fungal infection, by adding PsJN (1 × 10^6^ CFU/ml hydroponic medium) at each medium exchange.

### 2.3 Fungal growth conditions, infection and disease analysis

*Botrytis cinerea* strain 630 was grown on potato dextrose agar (PDA) plates at 22°C under indirect light. Conidia were collected from plates with sterile potato dextrose broth (PDB 12 g/l) and filtered to remove hyphae. Conidial concentration was adjusted to 1 × 10^6^ conidia/ml (Aziz et al., 2003). Collected conidia were used immediately or kept frozen at -80°C until use. For plant infection, germinated spores incubated at 22°C for 18–22 h were used. Two weeks after bacterial inoculation, plants were infected with 20 µl of germinated conidia per developed leaf. To aid in infection, light intensity was lowered to 60-80 μmol m^−2^s^−1^ and humidity increased to 80-100%. To provide high humidity necessary for *B. cinerea* infection, plants were held in closed transparent boxes one day before, and another day after the pathogen challenge. Also, aeration of roots in hydroponics was withheld during the two days of treatments. To assess the progression of pathogen attack, the average size of disease lesions developed four days post infection was measured and analyzed using image analysis software ImageJ (Schneider et al., 2012). For the analysis of gene expression, to obtain a homogeneous application, germinated conidia suspension was applied to the whole leaf areas. PDB was used in the same manner as a mock treatment.

### 2.4 Sampling

Roots and leaves of plants grown hydroponically were sampled at 24 hours post fungal infection (hpi), weighed, and immediately frozen in liquid nitrogen and stored at -80C until RNA extraction. Roots were also washed 2–3 times with sterile distilled H_2_O before freezing. Three replicates were collected for each treatment, each replicate comprising three individual plants. Treatments were: plants mock inoculated and mock infected - CNI, plants mock inoculated and infected with *B. cinerea* - CBc, plants inoculated with PsJN and mock infected - PNI, plants inoculated with PsJN and infected with *B. cinerea* - PBc.

### 2.5 RNA extraction

Leaf or root tissue samples collected at 24hpi were ground using mortar and pestle. 50mg of powder was used for RNA extraction. RNA was extracted with microRNA Purification Kit (Norgen BioTek) following protocol for plants. Both small and long RNA isolates were treated with the Ambion^®^ DNA-free™ DNase Treatment and Removal DNA kit. As total RNA was required as an input for RNA sequencing, small and long RNA fractions were combined. Three samples per treatment group per tissue were used for RNAseq (24 samples) as well as sRNAseq (24 samples).

### 2.6 mRNA library construction and sequencing

Twenty-four samples were sent for RNA sequencing performed by Novogene Bioinformatics Technology Co., Ltd. (Cambridge, UK). Integrity and purity of RNA was checked with Agilent 2100 Bioanalyzer, samples passed quality control were used for mRNA library construction. Library preparation included poly A enrichment and cDNA synthesis. Prepared libraries were sequenced on NovaSeq X Plus Series platform (Illumina, Inc., San Diego, CA, USA) using paired-end 150 bp sequencing strategy.

### 2.7 Bioinformatics analysis

The sequencing results were analyzed with standard Novogene pipeline. Clean data (clean reads) was obtained from raw data using fastp software. Paired-end cleaned reads were aligned to the reference genome using Hisat2 (v 2.0.5) (Mortazavi et al., 2008). *Arabidopsis thaliana* Genome assembly TAIR10.1 (ncbi_arabidopsis_thaliana_gcf_000001735_4_tair10_1) was used as the reference genome. The mapped reads of each sample were assembled by StringTie (v1.3.3b) (Pertea et al., 2015). Quantification of gene expression level was performed using FeatureCounts v1.5.0-p3 (Liao et al., 2014). DESeq2 (1.20.0) was used to analyze differentially expressed genes (DEGs) (Love et al., 2014). Genes with an adjusted P-value ≤ 0.05 found by DESeq2 were assigned as differentially expressed. Gene Ontology (GO) enrichment analysis (Young et al., 2010) of differentially expressed genes was implemented by the clusterProfiler R package (v 3.8.1), in which gene length bias was corrected. GO terms with corrected P-value less than 0.05 were considered significantly enriched by differential expressed genes.

Additionally, MapMan software was used to provide a general overview and graphic representation of DEGs involved in metabolic pathways and other biological processes (Thimm et al., 2004).

### 2.8 Small RNA library construction and sequencing

Twenty-four samples (twelve from leaves and twelve from roots) were sent to Novogene Bioinformatics Technology Co., Ltd. (Cambridge, UK) for small RNA sequencing. After adapter ligation to both ends of small RNA, first strand cDNA was synthesized during hybridization with reverse transcription primer. The double-stranded cDNA library was generated through PCR enrichment. After purification and size selection, libraries with insertions between 18~40 bp were ready for sequencing. The library was checked with Qubit and real-time PCR for quantification and Agilent 2100 Bioanalyzer for size distribution detection. Single-end 50-bp sequencing of small RNA libraries was performed on Illumina NovaSeq 6000 platform.

### 2.9 Small RNA bioinformatics analysis

The sequencing results were analyzed with standard Novogene pipeline for small RNAs. Clean reads were used to map small RNA to reference genome using bowtie (Langmead et al., 2009). To analyze small RNA expression and distribution on the reference sequence mapping was done without mismatch or with only one mismatch. Mapped small RNA tags were used to look for known miRNA. miRBase22.0 was used as reference, modified software mirdeep2 (Friedländer et al., 2012) and srna-tools-cli were used to obtain the potential miRNA and draw the secondary structures. Predicting the target gene of miRNA was performed by psRobot_tar in psRobot (Wu et al., 2012). Quantification of miRNA expression levels were estimated by TPM (transcript per million) through the following criteria (Zhou et al., 2011): Normalization formula: Normalized expression = mapped readcount/Total reads * 1000000. Differential expression analysis of two conditions/groups was performed using the DESeq2. The P-values were adjusted using the Benjamini & Hochberg method. Corrected P-value of 0.05 was set as the threshold for significantly differential expression by default. GO enrichment analysis was used on the target gene candidates of differentially expressed miRNAs.

The miRNA-mRNA network was constructed by using Cytoscape 3.10.1 software (Shannon et al., 2003).

## 3 Results

### 3.1 *Paraburkholderia phytofirmans* PsJN protects *Arabidopsis thaliana* against *Botrytis cinerea*

**Figure 1:**
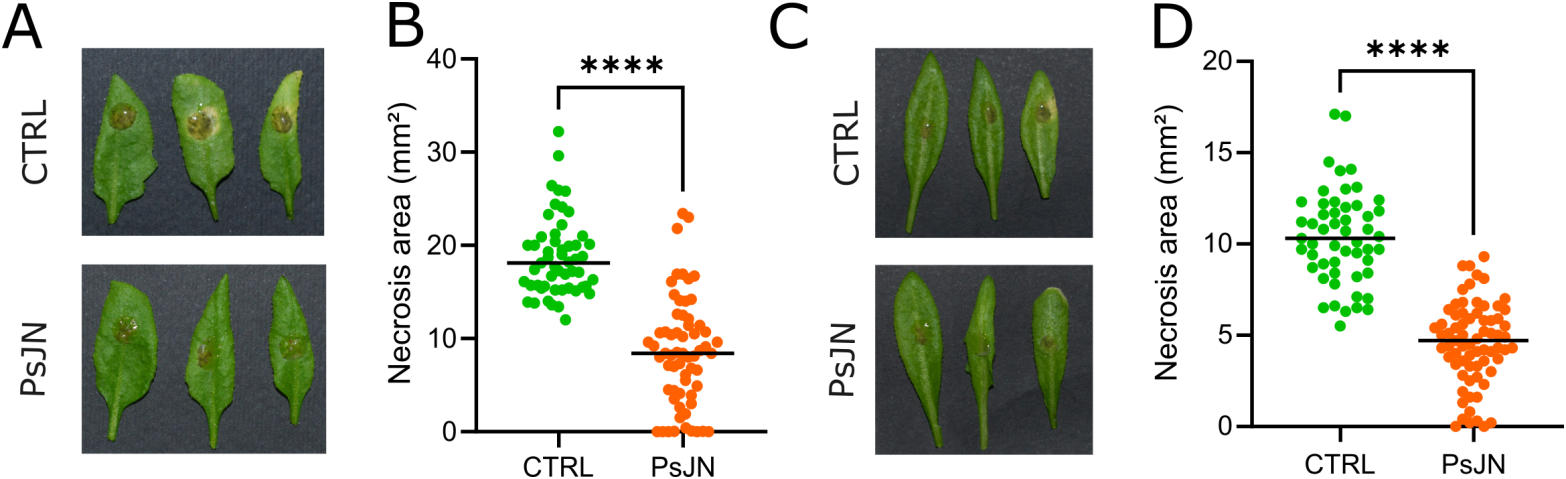
*Paraburkholderia phytofirmans* PsJN protects *Arabidopsis thaliana* against *Botrytis cinerea* and promotes plant growth. Representative photographs of Arabidopsis leaves infected with *B. cinerea* previously mock-inoculated or inoculated with PsJN; plants grown in soil **(A)** and hydroponics **(C)**. Necrosis area caused by *B. cinerea* in plants grown in soil **(B)** and hydroponics **(D)**. **** indicate significant difference at *P* < 0.0001 as determined by Mann-Whitney test analysis.

To test whether the PGPB *P. phytofirmans* PsJN induces a protective effect in *A. thaliana* challenged with *B. cinerea*, mature plants, previously inoculated with *P. phytofirmans* PsJN at the root level or mock-inoculated were infected with germinated spores of *B. cinerea*. Four days after infection, disease lesions were analyzed. Control plants (mock-inoculated) showed typical symptoms of necrosis, while *B. cinerea*-induced necrotic lesions in PsJN-inoculated plants were significantly smaller (P<0.0001). These results support the idea that PsJN triggered a systemic resistance against *B. cinerea*. This PsJN-induced resistance was observed in *A. thaliana* grown in both soil and hydroponics (**Figure 1**).

Aside from increasing *A. thaliana* resistance to *B. cinerea*, PsJN promoted growth of inoculated plants by increasing both leaf and root biomass (**Supplementary Figure S1**).

### 3.2 Transcriptome profiling of the PsJN-inoculated Arabidopsis plants

Recent advancements in next-generation sequencing have resulted in a surge of transcriptomic research examining alterations in host gene expression during interactions between plants and plant growth-promoting bacteria (PGPB). We performed RNA-Seq for the RNA isolated from roots and leaves of both PsJN-inoculated and non-inoculated Arabidopsis. Plants were grown in hydroponics to facilitate analysis of root transcriptome, alongside with the transcriptome of leaves.

**Figure 2:**
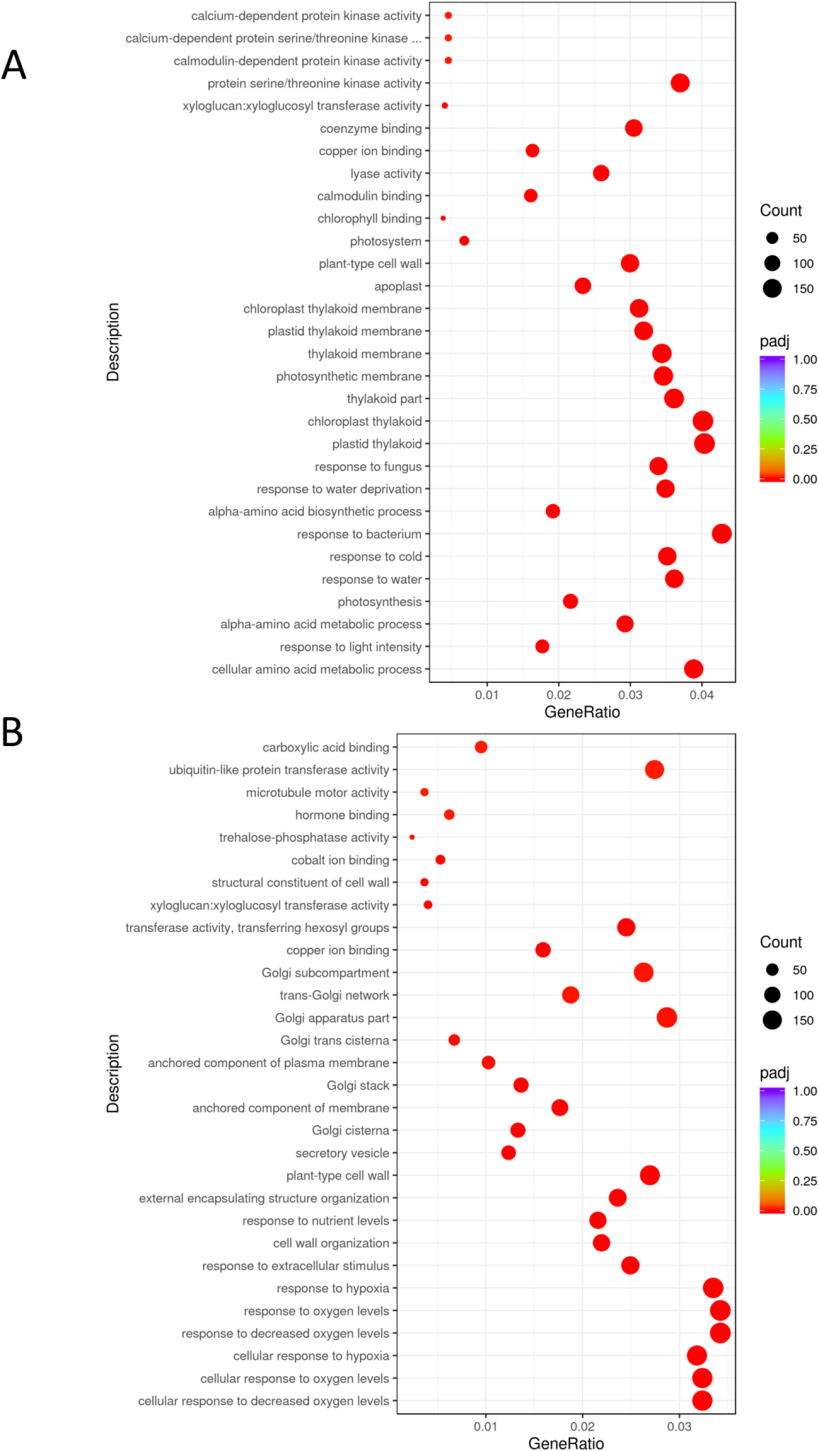
Gene Ontology (GO) enrichment analysis of DEGs in Arabidopsis induced by PsJN inoculation (comparison PNI *vs* CNI). GO enrichment chart displays the top 30 most significant pathways comprising genes with significantly changed expression in **(A)** leaves and **(B)** roots. The colors represent different levels of significance, with red indicating highly significant terms. padj: adjusted p-value.

Before *B. cinerea* infection, transcriptomic analysis showed a total of 3388 genes upregulated and 3346 genes downregulated in Arabidopsis roots inoculated with PsJN, compared to the non-inoculated control (comparison PNI *vs* CNI, found by DESeq2 with adjusted p-value ≤ 0.05). The number of differentially expressed genes (DEGs) showing upregulation of FC > 1.5 was 1746, and the number showing downregulation of FC < 0.6 was 2468. In leaves of PsJN-inoculated plants, 2573 were upregulated and 2438 downregulated compared to the non-inoculated control. The number of DEGs showing upregulation of FC > 1.5 was 598, and the number showing downregulation of FC < 0.6 was 514 (**Figure 2**; **Supplementary Table S1**).

At 24h post-infection with *B*. *cinerea*, 507 genes were upregulated and 474 genes were downregulated in Arabidopsis roots inoculated with PsJN, compared to the non-inoculated plants (comparison PBc *vs* CBc). The number of DEGs showing upregulation of FC > 1.5 was 81, and the number showing downregulation of FC < 0.6 was 172. In *B. cinerea*-infected leaves of PsJN-treated plants, 150 genes were upregulated and 224 were downregulated compared to the non-inoculated plants. The number of DEGs showing upregulation of FC > 1.5 was 55, and the number showing downregulation of FC < 0.6 was 115 (**Supplementary Table S1**).

**Figure 3:**
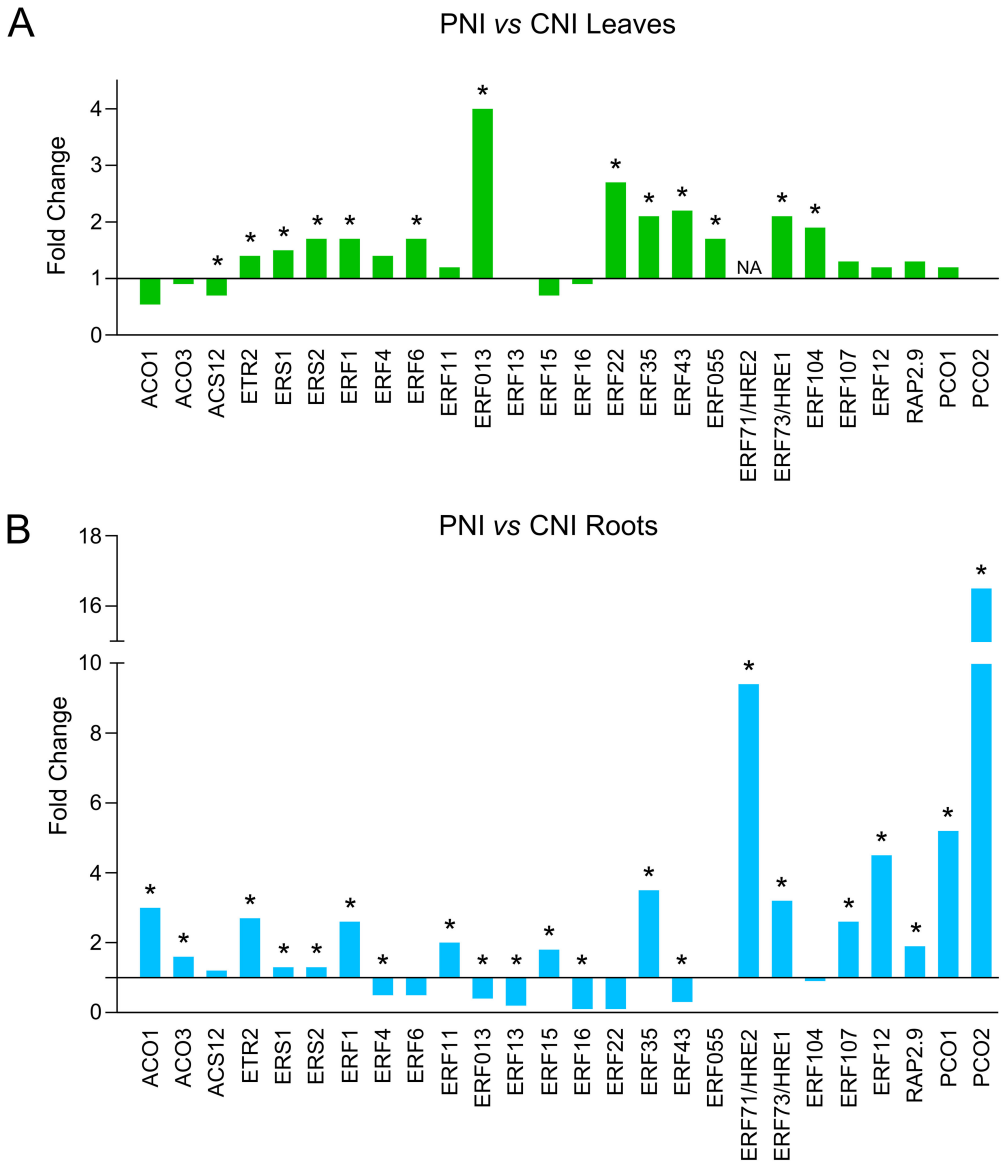
Expression levels of genes involved in ethylene synthesis and signaling pathway in roots and leaves of PsJN-treated plants before *B cinerea* inoculation. **(A)** Leaves of PsJN-treated plants *vs* non-inoculated control. **(B)** Roots of PsJN-treated plants *vs* non-inoculated control. * denotes values with adjusted P-value < 0.05.

Transcriptome analysis of plant roots and leaves before *B. cinerea* infection (PNI *vs* CNI) revealed several DEGs involved in responses to both abiotic and biotic stresses (**Figure 2**; **Supplementary Table S1**). Data indicated that PsJN modulates the expression of a number of genes involved in ethylene synthesis and signaling pathway in both leaves (**Figure 3A**) and roots (**Figure 3B**). PsJN induces a significant upregulation of *ACO1* and *ACO3* genes, encoding 1-aminocyclopropane-1-carboxylic acid oxidase in the roots (**Figure 3B**). Two other genes involved in ethylene perception like Ethylene Response *ETR2* and Ethylene Response Sensor *ERS2* were also upregulated in both leaves and roots of plants inoculated with PsJN. The transcriptome analysis also revealed that PsJN differently affects the expression of numerous ethylene response factors ERFs, depending on their target genes and the plant organ (**Figure 3**). The most of ERF genes are upregulated in leaves, while in roots both up and downregulation of different ERF genes is detected as a response to PsJN inoculation.

### 3.3 PsJN modulates the expression of genes related to abiotic stress responses

**Figure 4:**
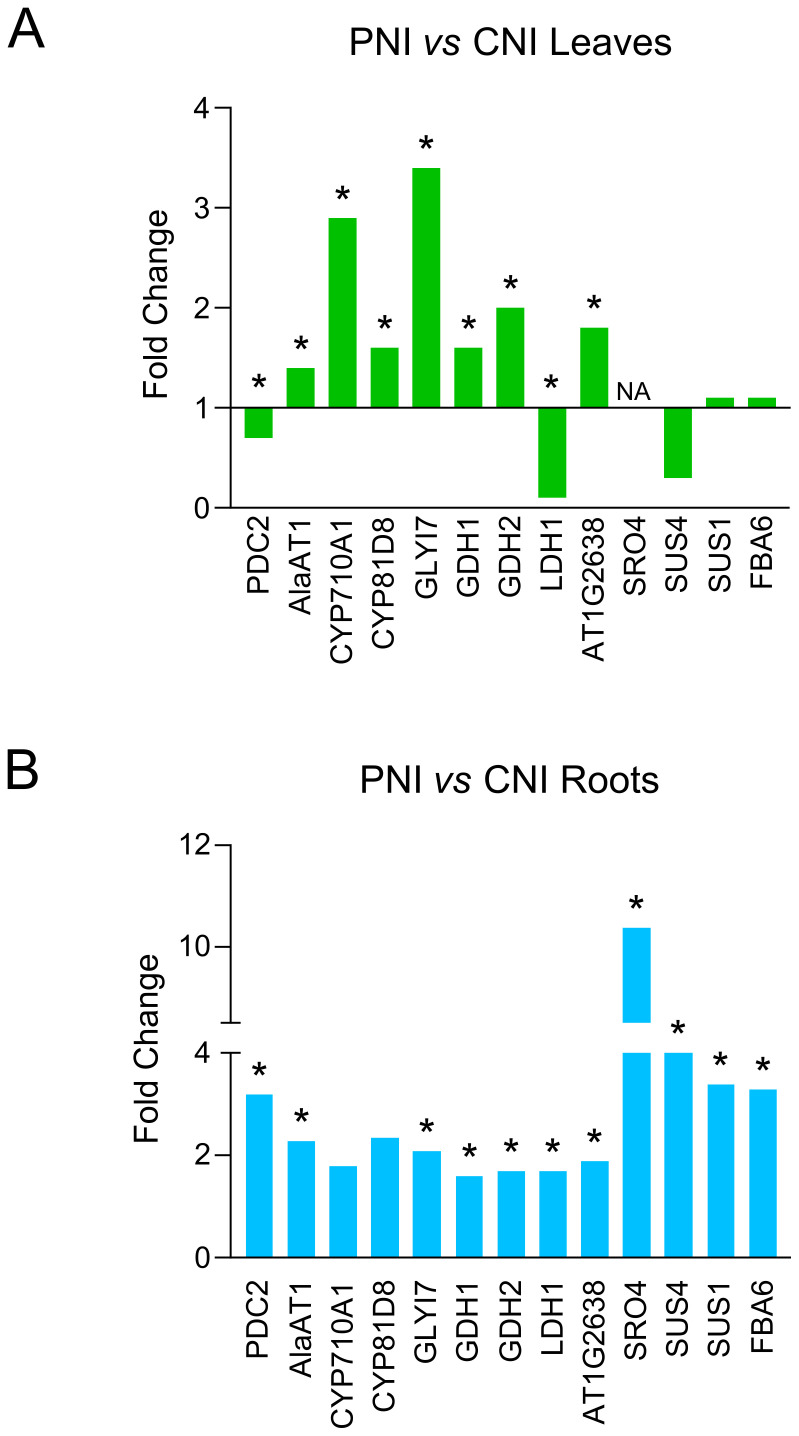
Expression levels of genes involved in hypoxia response in roots and leaves of PsJN-treated plants before *B. cinerea* inoculation. **(A)** Leaves of PsJN-treated plants *vs* non-inoculated control. **(B)** Roots of PsJN-treated plants *vs* non-inoculated control. * denotes values with adjusted P-value < 0.05.

Gene Ontology of the PsJN-upregulated genes indicated functional categories of biological processes including pathways related to responses to abiotic stimuli, decreased oxygen levels, nutrient levels and absence of light (**Figure 2**). We identified a significant number of genes related to hypoxia sensing and response in both roots and leaves (**Figures 3**, **4**). This could be related to hydroponic plant growth conditions without aeration of roots during 48h of treatment or to the pre-exposure of the plants to high humidity before *B. cinerea* infection. The key oxygen sensors are members of the ERFVIIs family: Hypoxia-Responsive ERFs (HRE) and Related to Apetala2 (RAP2) (Licausi et al., 2010; Gibbs et al., 2011). The expression of *HRE1* and *HRE2* increased significantly in the roots of Arabidopsis grown with PsJN, while *HRE1* was upregulated in the leaves. PsJN did not significantly affect the expression of key RAP transcription factors, with only *RAP2.9* being upregulated twofold in the roots. *PCO1* and *PCO2* (encoding plant cysteine oxidases), which also act as oxygen sensors and are involved in the N-end rule pathway, were also upregulated in roots inoculated with PsJN.

Analyzing transcriptome sequencing in the roots, we also found that PsJN significantly upregulated transcription of some genes involved in fermentation processes using pyruvate as a starting substrate, including *PDC2* (pyruvate decarboxylase2), *LDH1* (lactate dehydrogenase), and *AlaAT1* (alanine aminotransferase). We also noticed a significant increase of other genes important for plant survival under low-oxygen conditions, such as *GDH1* and *GDH2* (glutamate dehydrogenase), *SUS1* and *SUS4* (encoding sucrose synthases), *FBA6* (fructose-1,6-bisphosphate aldolase 6), an enzyme involved in glycolysis and gluconeogenesis and *GLI17* (glyoxalase I) (**Figure 4**).

### 3.4 PsJN modulates the expression of genes associated with biotic stress responses

**Figure 5:**
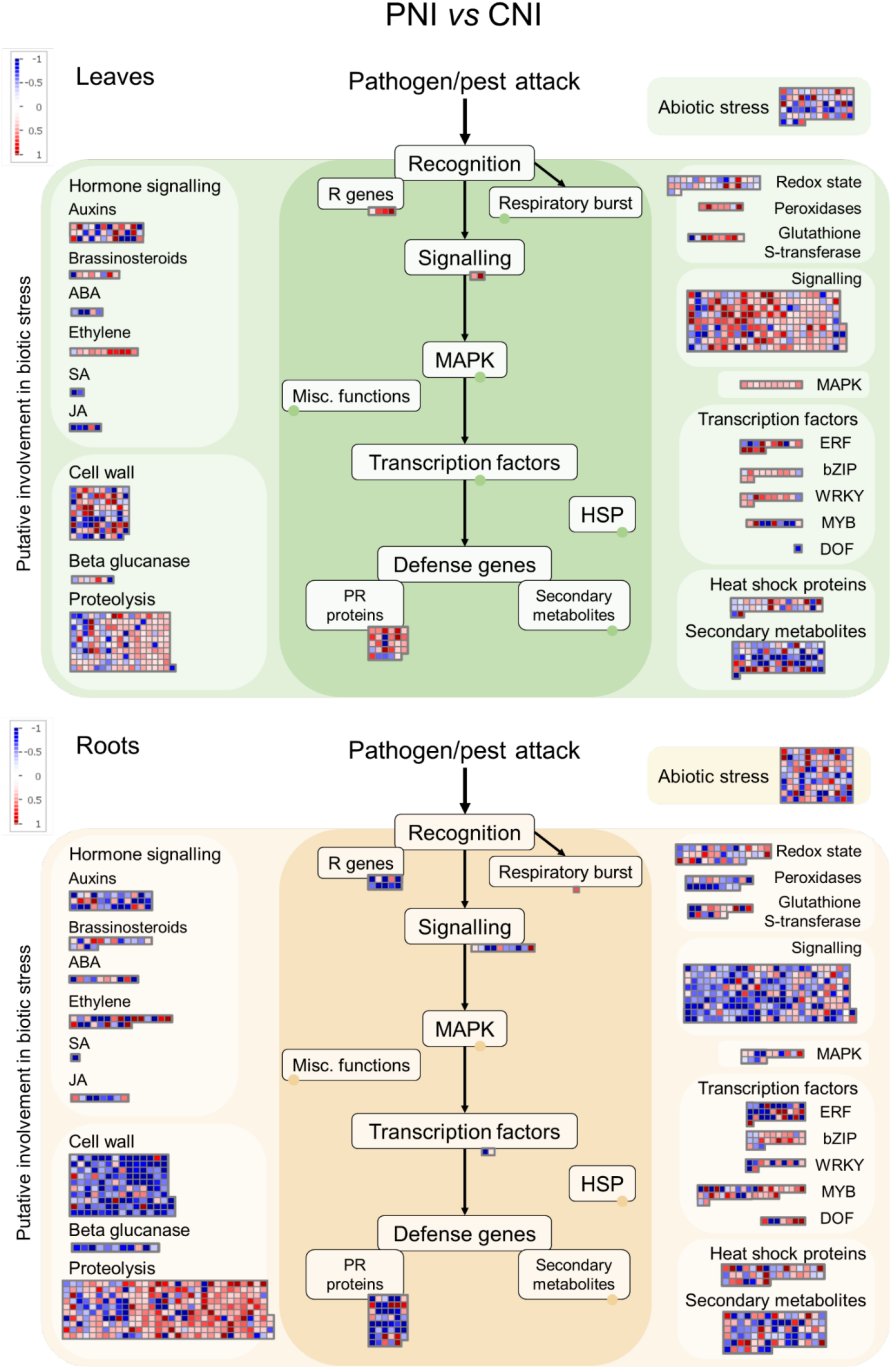
MapMan analysis of genes differentially expressed in roots and leaves of Arabidopsis in response to PsJN before *B. cinerea* infection. The green panels indicate leaves, while the beige panels represent roots.

**Figure 6:**
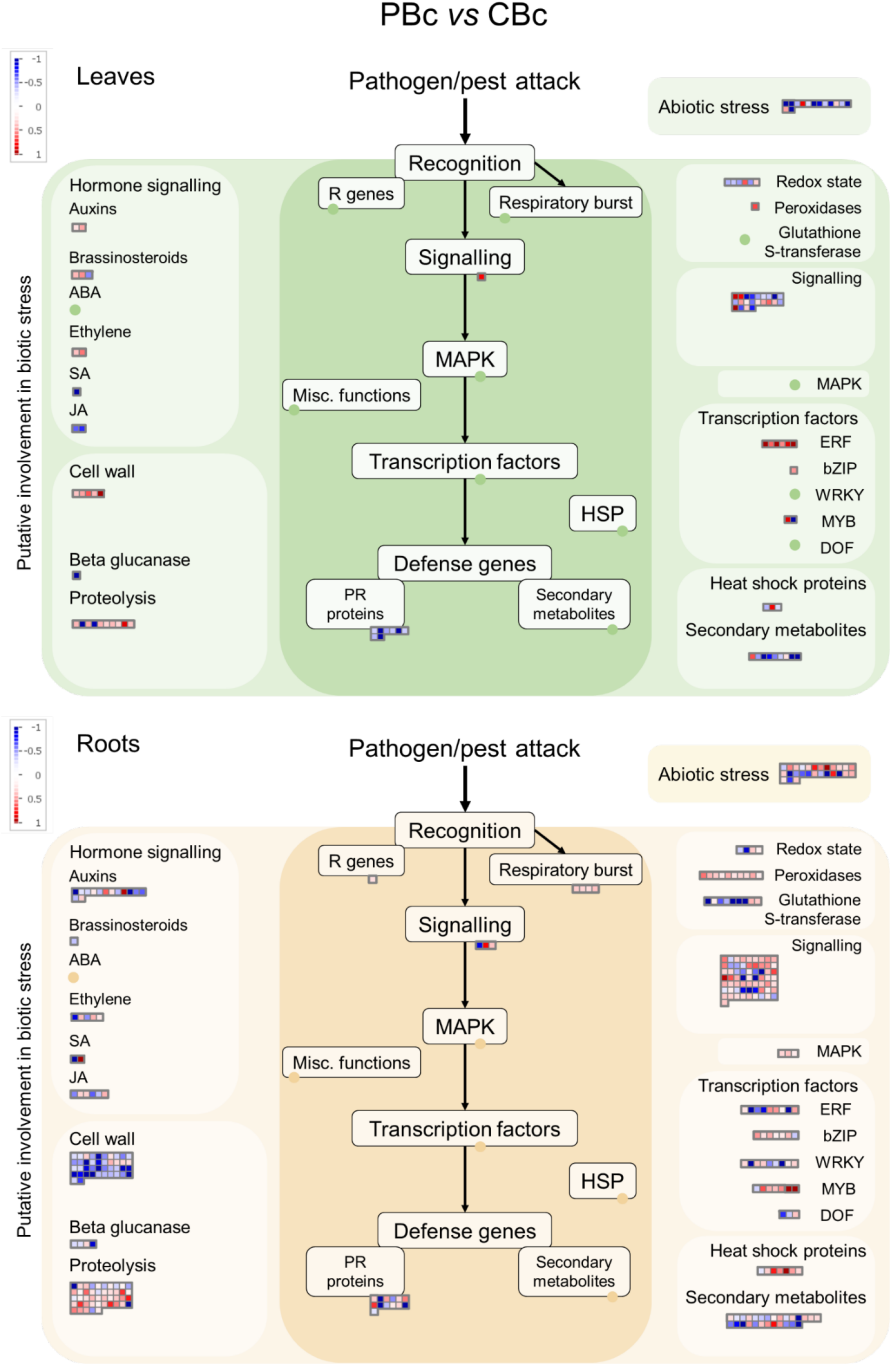
MapMan analysis of genes differentially expressed in in roots and leaves of Arabidopsis response to PsJN 24 hours after *B. cinerea* infection. The green panels indicate leaves, while the beige panels represent roots.

Using the MapMan software (Thimm et al., 2004), we obtained an overview of the PsJN-induced changes in pathways involved in biotic stress. As shown in **Figures 5**, **6**, the PsJN-induced change in *A. thaliana* transcriptome was much more pronounced prior to *B. cinerea* infection than in plants 24 hours after *B. cinerea* infection (24hpi). Before infection (**Figure 5**; **Supplementary Tables S2**, **S3**), the most noticeable changes induced by PsJN were in genes related to signaling, proteolysis and cell wall. While in roots the most of the signaling-associated genes (like E3 ubiquitin-protein ligases and proteases of different types) were downregulated, in leaves PsJN inoculation led to an opposite effect. PsJN altered the expression level of transcription factors involved in biotic stress responses belonging to ERF, bZIP, WRKY, MYB and DOF families. Genes related to phytohormone signaling pathways were also affected by bacterial inoculation. There is a noticeable tendency for upregulation of genes involved in ethylene pathway in leaves in similar way as for ERF transcription factors, while in roots the expression of different genes involved in ethylene regulation were differently altered. Interestingly, while both up and downregulation of cell wall-related genes was observed in leaves, in roots PsJN predominantly led to a decreased level of their expression. A prominent influence of PsJN was noticed on the expression of genes involved in proteolysis in both roots and leaves, more conspicuously, though, in roots. Pathogenesis related (PR) genes were mostly downregulated in PsJN inoculated roots, while more upregulated *PR* genes were found in leaves of the same plants.

Among the much smaller number of PsJN-influenced genes in plants 24 hours after *B. cinerea* infection, we still notice stimulation of *ERF* transcription factors in leaves (**Figure 6**; **Supplementary Tables S4**, **S5**). Also, although fewer cell wall-related genes were affected they are predominantly decreased in roots, while some of the genes were upregulated in leaves.

### 3.5 Small RNA analysis

To explore whether PsJN inoculation influences miRNA expression in *A. thaliana* under hydroponic conditions and during *B. cinerea* infection, small RNA from leaves and roots, in the four different treatments, were sequenced. An average of 30 million clean reads was generated for each sRNA library. Of that, 19.2 million reads were 18–30 nt in length. Small RNA identified as micro-RNA were further analyzed.

### 3.6 Identification of known and potentially novel miRNAs

In leaves, a total of 307 miRNAs were detected belonging to 109 miRNA families, while 28 of detected miRNAs were novel miRNAs. In roots, a total of 228 miRNAs belonging to 96 miRNA families were detected during analysis, 15 of which were novel miRNA. Families of miRNAs with most miRNAs detected were MIR156 and MIR159 in both leaves and roots.

### 3.7 miRNA expression patterns

Micro RNA expression was barely affected by PsJN inoculation, in both non-infected and *B. cinerea*-infected plants (**Supplementary Table S6**).

In non-infected leaves only one miRNA (ath-miR8183) was significantly upregulated (more than four-fold). Predicted targets of this miRNA are related to protein degradation and DNA synthesis. In roots, there were no miRNAs with significantly changed expression.

During *B. cinerea* infection, PsJN inoculation led to a significant change of only two miRNAs in leaves. Ath-miR163 and ath-miR845a were downregulated; ath-miR845a was lowered more than two-fold, while ath-miR163 was affected to a lesser extent. Putative targets of these miRNAs are related to phytohormone metabolism (especially for salicylic acid, SA), DNA synthesis, posttranslational modification of proteins and cell division. In roots, only one miRNA (ath-miR161.1) was upregulated and two miRNAs (ath-miR158a-3p and ath-miR159c) were downregulated. Genes involved in transcription regulation were recognized as the potential targets of these miRNAs.

To get an insight into the changes in the miRNA expression pattern induced by *B. cinerea* infection, regardless of PsJN inoculation, we analyzed miRNAs differentially expressed in plants infected with *B. cinerea* (CBc) or mock infected (CNI). In leaves, twenty miRNAs were significantly (corrected P-value < 0.05) differentially expressed, half of them upregulated and the other half downregulated upon infection (**Supplementary Table S6**). Five of the upregulated miRNAs (ath-miR157a-3p, ath-miR161.2, ath-miR168b-3p, ath-miR169f-3p and ath-miR858b) and five of the downregulated (ath-miR161.1, ath-miR1888a, ath-miR5020b, ath-miR5634 and ath-miR863-5p) were more than two-fold changed. One miRNA ath-miR858b, was highly upregulated (more than eight-fold).

In roots, only one miRNA (ath-miR398b-5p) was significantly differentially expressed, but its downregulation in infected plants was very remarkable, more than 77-fold.

Predicted targets of miRNAs differentially expressed after *B. cinerea* infection are mainly involved in the regulation of transcription and protein synthesis and degradation. Putative target genes are also involved in development, stress responses, RNA processing, cell wall biogenesis and organization, regulation of cellular component biogenesis and phenylpropanoid metabolic process. Targets of ath-miR398b-5p, which is highly downregulated in roots, are mainly involved in RNA regulation of transcription, transport and signaling.

*B. cinerea* infection also affected miRNA expression in plants previously inoculated with PsJN (PBc vs PNI). Twenty miRNAs were differentially expressed in leaves, while there were no significantly changed miRNAs in roots (**Supplementary Table S6**). One of the upregulated miRNAs, ath-miR398b-5p was highly expressed, more than eight-fold, while the expression of ath-miR157a-3p, ath-miR161.2, ath-miR167c-3p, ath-miR169f-3p and ath-miR824-5p was decreased more than two-fold. Other miRNAs, including ath-miR5020b, ath-miR5634 and ath-miR8183 were also downregulated more than two-fold in the leaves of PsJN inoculated plants after *B. cinerea* infection.

Predicted targets of miRNAs altered in response to *B. cinerea* infection (PBc vs PNI), are involved in signaling, defense, transcription regulation, proteolysis and hemicellulose synthesis.

### 3.8 Co-expression analysis of *miRNA-mRNA pairs*

To investigate miRNA-mRNA regulatory mechanisms, we performed an integrative analysis of transcriptome and small RNA sequencing data. A total of 487 genes were predicted as targets of differentially expressed miRNAs. Most miRNAs were predicted to target multiple genes, with both positive and negative miRNA-mRNA interactions observed. For example, the upregulated ath-miR396b-5p was shown to downregulate five of its targets, while three targets were upregulated.

**Figure 7:**
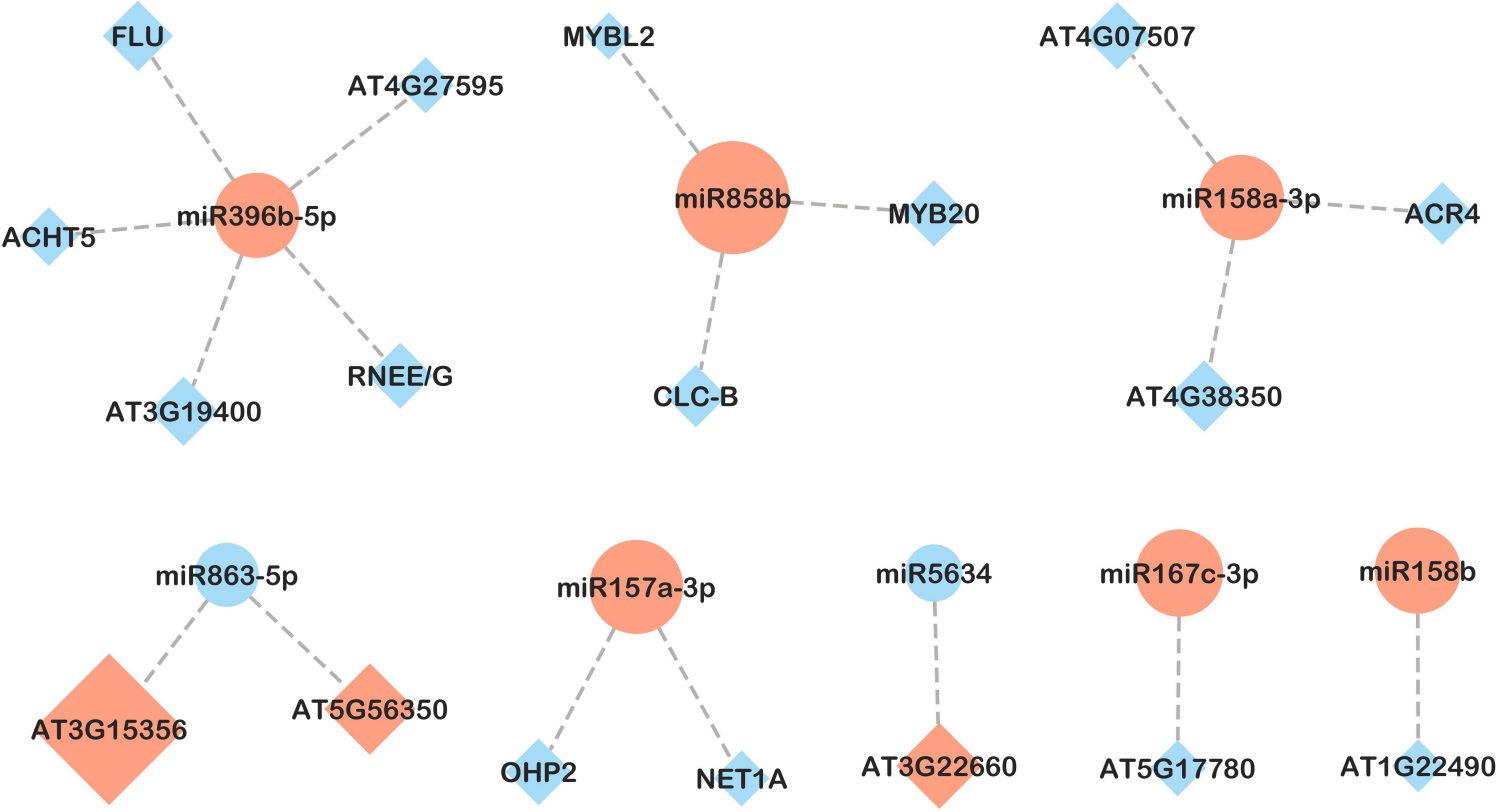
miRNA-mRNA network in leaves in response to *B. cinerea* infection (CBc *vs* CNI comparison). Candidate miRNA-mRNA target pairs including 8 differentially expressed miRNAs and 18 differentially expressed genes were integrated in the network. Diamonds represent mRNAs and circles represent miRNAs. Upregulated and downregulated genes are colored in red and blue, respectively. The size of the pattern represents the fold change in expression between CBc and CNI (miRNA-mRNA pairs with only inverse expression pattern are shown).

**Figure 8:**
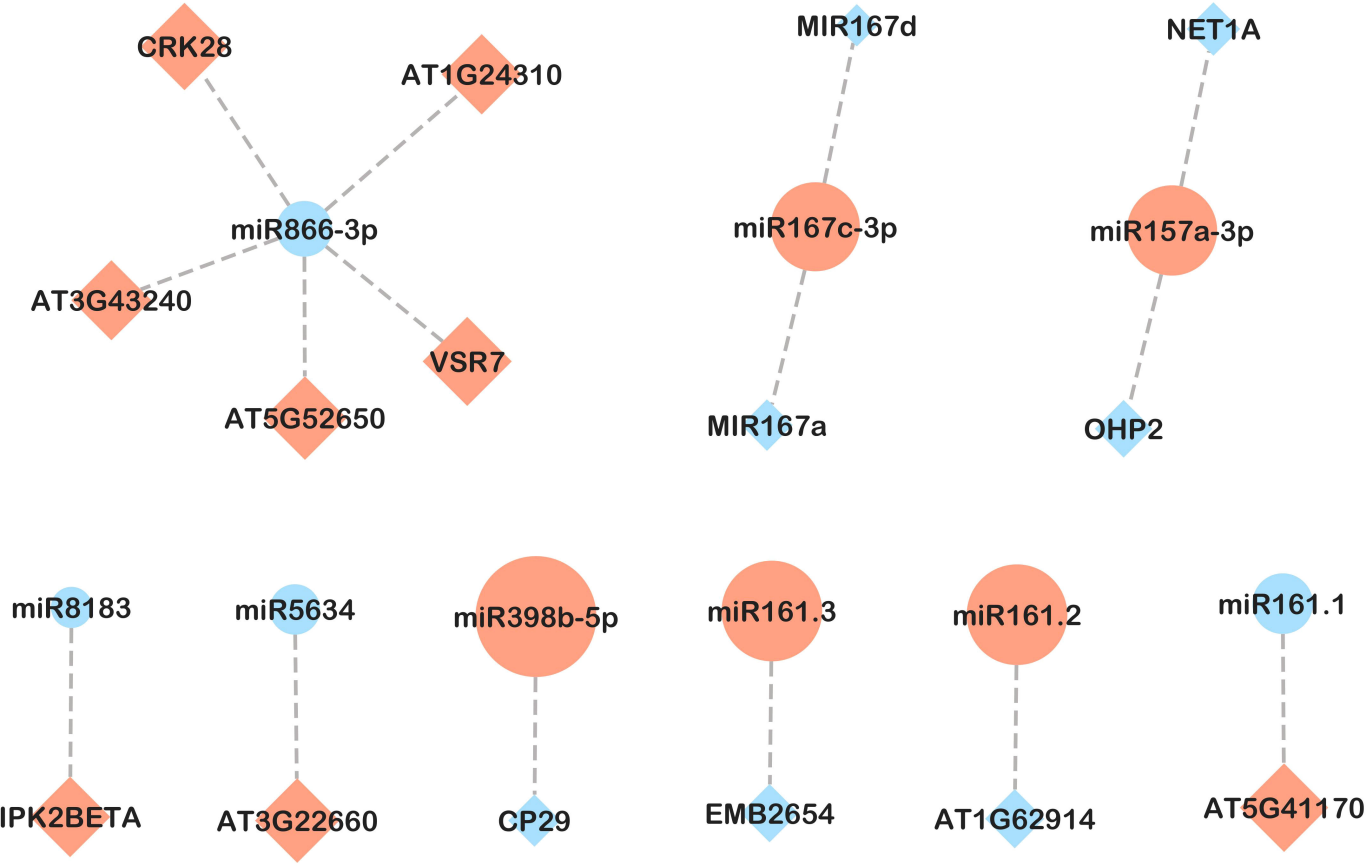
miRNA-mRNA network in leaves in response to *B. cinerea* infection in plants inoculated with PsJN (PBc *vs* PNI comparison). Candidate miRNA-mRNA target pairs including 9 differentially expressed miRNAs and 15 differentially expressed genes were integrated in the network. Diamonds represent mRNAs and circles represent miRNAs. Upregulated and downregulated genes are colored in red and blue, respectively. The size of the pattern represents the fold change in expression between PBc and PNI (miRNA-mRNA pairs with only inverse expression pattern are shown).

In leaves, of 108 miRNA-mRNA pairs examined, 30 exhibited opposite gene expression patterns, all of which were detected in response to *B. cinerea* (CBc vs CNI and PBc vs PNI treatment comparisons, **Figures 7**, **8**). In both comparisons, higher expression of ath-miR157a-3p was associated with lower expression of two target genes: NET1A (KIP1-like protein MYB-related) and OHP2 (ONE-HELIX PROTEIN2). In the CBc vs CNI comparison (**Figure 7**), ath-miR168a-3p and its target gene BDG4 (lysophospholipase BODYGUARD 4) showed an inverse expression pattern. The integrated analysis also revealed possible interactions between miRNAs and transcription factors. For example, ath-miR858b was highly expressed in the CBc treatment, while its targets belonging to the MYB family of transcription factors were downregulated. Similarly, an inverse expression of ath-miR158b and its target, the transcription factor bHLH94 (basic Helix-Loop-Helix), was observed under the same treatment.

Regarding response to *B. cinerea* infection in plants inoculated with PsJN (PBc *vs* PNI, **Figure 8**), an inverse expression pattern was shown for ath-miR161.1 and ath-miR161.2 and members of the pentatricopeptide repeat (PPR-like) superfamily of proteins. Of the six predicted targets of ath-miR398b-5p, only CP29A (chloroplast RNA-binding protein 29) exhibited an inverse expression pattern. Additional miRNA-target pairs with inverse expression trends included ath-miR5634 and rRNA-processing protein EBP2, as well as ath-miR8183 and inositol polyphosphate kinase 2 beta (Ipk2β). Notably, we detected a negative correlation between the expression of ath-miR866-3p and its predicted target, an antifungal-related gene CRK28, a cysteine-rich receptor-like protein kinase (RLK).

## 4 Discussion

The beneficial bacteria belonging to genera *Pseudomonas, Burkholderia* and *Paraburkholderia* were previously shown as effective in controlling the necrotrophic fungus *B. cinerea* in some host plants, including Arabidopsis (Nie et al., 2017), cucumber (Zhu et al., 2021), and grapevine (Miotto-Vilanova et al., 2016). In this study, we examined the efficiency of *P. phytofirmans* PsJN in inducing systemic resistance to *B. cinerea* and promoting plant growth in *A. thaliana*. Our data also support the capacity of PsJN to reduce *B. cinerea* symptoms and improve plant growth when it was applied at the root level of *A. thaliana* grown both in soil and in hydroponics (**Figure 1**). This protective effect indicates that PsJN triggers systemic resistance against *B. cinerea* without compromising plant fitness. These beneficial effects could be related to a PsJN-induced priming state of the plants. However, insights about the mechanisms involved in this resistance state remain unclear so far. In this study, we used RNA-Seq sequencing and hydroponic system to investigate changes in gene expression in both roots and leaves, since most of the previous studies dealt only with processes occurring in leaves. We showed that PsJN induces transcriptional and miRNA reprogramming both locally (in roots) and systemically (leaves) before and after *B. cinerea* infection.

### 4.1 PsJN modulates the expression of abiotic stress response genes

The comparative transcriptome GO analysis of the PsJN-inoculated and control plants, revealed upregulation of pathways related to responses to abiotic stimuli, low oxygen levels, nutrient levels and low light intensity (**Figure 2**). Among the DEGs related to abiotic stresses we identified a significant number of genes related to hypoxia response. PsJN induced alterations in genes involved in oxygen sensing and signaling, hypoxia-related transcription regulators and some of the effector genes required for hypoxia response and tolerance (**Figures 3**, **4**). This could be related to hydroponic plant growth conditions or to the pre-exposure of the plants to high humidity before *B. cinerea* infection. Ethylene has been shown to play a significant role in helping plants to cope with hypoxia, particularly during flooding stress (Liu et al., 2022). The expression patterns of genes associated with ethylene synthesis, sensing and signaling were modulated by PsJN inoculation in hydroponic conditions. PsJN affected the expression of numerous ERFs (Ethylene Responsive Factors), known as regulators of various ethylene-responsive genes and other important functions linked to abiotic stresses (**Figure 3**). Also, enzymes involved in plant adaptation to low oxygen conditions (PDC2, LDH1, AlaAT1, GLYI7, SRO4, FBA6, SUS1 and SUS4) were found upregulated, suggesting activation of alternative metabolic pathways for energy production and maintaining cellular functions (**Figure 4**).

Overall, PsJN inoculation positively influences various genes and pathways involved in response to hypoxia. It improves ethylene pathways and activates fermentation pathways to sustain energy production and optimize metabolic processes.

### 4.2 PsJN modulates expression of genes involved in microbe-plant interactions and ISR

Although most beneficial bacteria evade and suppress the local immune response to successfully colonize plant tissues, they modulate systemic plant defenses in a complex way to fight subsequent pathogens through induced systemic resistance (ISR). In this study, while the transcriptome was significantly altered in response to PsJN in non-infected plants, we detected only limited number of PsJN-modulated genes after 24 hours of *B. cinerea* infection (**Figure 6**; **Supplementary Tables S4**, **S5**). Although no necrotic lesions were visible at that time, gene expression had already been changed tremendously as a response to the pathogen challenge. Nevertheless, the increased expression of defense-related genes in the leaves prior to infection, combined with the protective effect of PsJN visible at later stages of infection, indicates that bacterial inoculation primes the plants for subsequent pathogen attack.

The most noticeable changes induced by PsJN in *A. thaliana* in our experiments, were in genes related to signaling, ethylene pathway, proteolysis and cell wall (**Figure 5**). Perception of microbes and signal transduction are crucial for a plant’s ability to shape its microbiome and to distinguish beneficial from pathogenic microorganisms. Among the genes involved in signaling, PsJN influenced a considerable number of receptor kinases and calcium binding proteins (**Supplementary Tables S2**, **S3**). Owing to their great diversity, receptor-like kinases (RLKs) regulate a wide range of vital processes in plants, including development and abiotic and biotic stress responses (Jose et al., 2020). We found both increased and decreased signaling genes in aboveground as well as in underground plant parts; however, they were predominantly down-regulated in roots, while mostly being upregulated in leaves. It is interesting to note that a loss of a plant receptor kinase recruits PGPB *Pseudomonas fluorescens* in Arabidopsis roots (Song et al., 2021). Although mutualists and pathogens share some of the microbe-associated molecular patterns (MAMPs) recognized by RLK, there are also epitopes specific to certain microbes. By combining the perception of different epitope variants, effectors, damage-associated molecular patterns (DAMPs) and other signals, plants can distinguish microorganisms and respond appropriately (Zhang and Kong, 2022). We can assume that the ability of a PGPB to modulate the expression of receptor kinases and other signaling genes, could facilitate colonization of the root, on the one hand, and alter the overall plant perception and response to microorganisms, on the other.

Effective plant immune reactions rely not only on *de novo* synthesis of defense proteins but also on their post-translational modifications and regulated degradation. Post-translational modifications enable rapid and dynamic responses to external stimuli and play an important role in plant immunity (Sharma et al., 2021). Increasing evidence supports a positive role of SUMOylation in plant immunity. This post-translational modification regulates the functioning of the plant immune response, since components of the defense signaling pathway are recognized as SUMO targets. On the other hand, pathogens target host SUMOylation to manipulate plant defense. A positive correlation between SUMOylation and fungal resistance has been found (Li et al., 2013a; Colignon et al., 2017). In our study, a remarkable increase in the expression of SAE1B, the SUMO-activating enzyme 1B (19-fold) was detected in leaves of PsJN-inoculated plants, suggesting a role of SUMOylation in the protective effect of PsJN against *B. cinerea* infection (**Supplementary Table S1**).

The ubiquitin–proteasome system (UPS) regulates all stages of plant immunity. On the other hand, pathogen effectors interfere with the plant UPS by hijacking its functions (Langin et al., 2023). Role of UPS in the establishment of mutualistic plant-microbe interactions is also proposed, however, studies addressing this question are very limited. It was shown that endophytic colonization of *A. thaliana* with a PGPB strain *Kosakonia radicincitans* interferes with ubiquitin-dependent protein degradation, while increasing the protein expression of 20S proteasome alpha-3 subunit (Witzel et al., 2017). Also, Dudnik et al. (2014) reported that endophyte *Rhizobium* sp. strain AP16 synthesizes a proteasome inhibitor syringolin A. Very complex interplay in plant-microbe interactions related to proteolytic pathway include microbe-induced targeting of immune proteins for degradation, as well as impairment of protein turnover required for adequate immune response (Langin et al., 2023). Transcriptome changes do not always reflect protein levels or their functionality; however, alterations in the expression of UPS and proteolysis related genes, provoked by PsJN inoculation, indicate that this strain modulates action of plant proteolytic pathway (**Figure 5**; **Supplementary Tables S2**, **S3**).

Among the defense-related genes, we detected a PsJN-dependent downregulation of pathogenesis-related (PR) proteins and beta glucanases in roots, while several PR genes were up or downregulated in leaves. These proteins are indispensable for plant defense and their expression increases when they encounter pathogenic invaders. Some studies reported an increased accumulation of PR proteins in PGPB-inoculated plants as part of the ISR mechanism of plant protection (van de Mortel et al., 2012; Leandro et al., 2019). However, others found PGPB-induced downregulation of PR proteins, and it has been suggested that suppression of some factors of the plant defense response may serve to facilitate colonization of plant tissues (Brusamarello-Santos et al., 2012; Thomas et al., 2019; Soares et al., 2024).

Both pathogenic and beneficial microorganisms have been found to influence cell wall composition, to facilitate their attachment and colonization. Downregulation of genes related to cell wall formation and modification has previously been observed in Arabidopsis and rice. It has been suggested that PGPB may suppress modifications of root cell walls, to support early stages of root colonization (Lakshmanan et al., 2013; Rekha et al., 2018). The effect of PGPB on root growth depends on bacterial strain and titer, plant species, developmental stage and growth conditions. In our experimental setup, most cell wall-related enzymes and proteins were downregulated in PsJN-inoculated roots. Transcriptome analysis was performed on plants several weeks after the first inoculation; however, it can be assumed that the hydroponic growth conditions and the lowered oxygen content that prevailed during the 48 hours before sampling also have an influence on the gene expression patterns.

While receptor kinases and calcium binding proteins, peroxidases, cell wall-related proteins and PR proteins, were mostly found downregulated in the PsJN-inoculated roots, their expression in leaves was predominantly upregulated (RLK, peroxidases and PR proteins), or differently regulated (for different cell wall proteins). Also, both increase and decrease of various transcription factors and hormone pathways related to biotic stimuli response were detected in PsJN-inoculated roots, while in leaves their upregulation is prevalent, especially regarding ethylene (ET) dependent pathway (**Figure 5**; **Supplementary Tables S2**, **S3**). These transcriptome alterations in leaves before occurrence of pathogen are in line with the PsJN-induced alleviation of disease symptoms after *B. cinerea* infection (**Figure 1**). They underline a priming state of plant for faster and stronger responses upon pathogen attack, i.e. ISR.

Both local and systemic changes in expression of ET pathway genes were detected in response to PsJN-inoculation (**Figure 3**). The altered expression of ET pathway genes in the roots during beneficial interactions suggests that ET signaling plays a role not only in defense against pathogens, but also in recognizing beneficial endophytic microbes, potentially regulating root colonization (Shekhawat et al., 2023). ET can act as both positive and negative regulator depending on the microbial strain and the stage of interaction. Some beneficial microbes require ET for plant growth promotion in stressful conditions (Shekhawat et al., 2021). On the other hand, some beneficial bacteria produce ACC deaminase, which lowers ET levels to alleviate plant stress and to promote plant growth by suppressing the ET-dependent plant defense system (Glick, 2014). This suggests that the effect of ET on plant-microbe interactions is concentration-dependent, and a finely regulated ET pathway is crucial for establishing beneficial interactions and enhancing protective effects (Shekhawat et al., 2023).

PsJN is found to both reduce ET level locally, through the activation of ACC deaminase (Sun et al., 2009), and to upregulate ET synthesis genes in both roots and shoots (Pinedo et al., 2015). It was also shown that plant growth promotion by PsJN is ET- dependent in Arabidopsis, since this effect was lost in ein2–1 mutants (Poupin et al., 2016). Thus, it is suggested that PsJN finely regulates ET to induce beneficial effects in plants. We found that in roots PsJN differently regulates different ET-related genes, while in leaves they were mostly upregulated. Taken together with the finding that PsJN mostly downregulated genes related to microbe recognition, signaling and PR in roots, we suppose that attenuation of immune response and modulation of ET pathway could serve to facilitate root colonization and the establishment of mutualistic relationship.

In leaves, the upregulation of ET pathway and signaling genes indicates PsJN-induced systemic response as a preparation of plant to the subsequent pathogen attack. It is also worth noting that after *B. cinerea* infection among a small number of DEGs observed in PsJN-inoculated plants we notice ET-related genes, all upregulated, indicating an important role of ET in the PsJN-mediated ISR in Arabidopsis. This is in accordance with the renowned importance of this phytohormone for plant resistance to necrotrophic pathogens (Ghozlan et al., 2020).

Along with ET, some PGPB were found to stimulate the JA pathway, which is required for defense-related gene expression in plants. We noticed that PsJN increases the expression of ERF1 which is known to integrate signals from both JA and ET pathways (Lorenzo et al., 2003). However, none of the enzymes involved in JA biosynthesis were found to be differentially expressed in PsJN-inoculated plants 24h after *B. cinerea* infection.

### 4.3 *B. cinerea* triggered transcriptome and sRNAome changes in Arabidopsis

Transcriptome analysis of Arabidopsis infected with *B. cinerea* provides valuable insights into the plant’s defense mechanisms and the pathogen’s infection strategies. As previously reported, the host plant undergoes extensive transcriptional reprogramming during *B. cinerea* infection (Wei et al., 2024). Our research confirmed transcriptional changes in metabolic pathways, signaling cascades, and transcriptional regulation in Arabidopsis plants after *B. cinerea* infection (**Figure 6**; **Supplementary Tables S4**, **S5**).

The understanding of how Arabidopsis roots respond to leaf *B. cinerea* infection is also of relevant importance for elucidating the complex signaling pathways that govern plant defense mechanisms, but was usually neglected. In our pathosystem almost 300 genes were significantly upregulated and about 600 downregulated in roots upon *B. cinerea* infection. They are involved in several important processes: ET biosynthesis and regulation, sugar metabolism and stress defense. Among the ET-related genes, the *ACO3* (1-Aminocyclopropane-1-Carboxylate Oxidase 3) gene exhibited the most significant difference in expression compared to non-infected plants. This gene plays a crucial role in the final step of ET biosynthesis in plants. ET-responsive transcription factors such as ERF15, ERF35, ERF73, RAP2.9, and ORA59 were also upregulated in roots, suggesting a key role of ET in the response to pathogen attack in both affected plant organs and distant below-ground parts. We also identified other upregulated genes involved in sugar metabolism in response to *B. cinerea* infection, such as SUS3 (sucrose synthase), and SWEET11 and SWEET12 (sugar transporters). It has previously been reported that SWEET11 and SWEET12 transporters accumulate in the roots under stress conditions, facilitating the unloading of sucrose from the apoplast to the sink cells in the roots (Fatima et al., 2022). This process promotes root growth by reallocating more sucrose from the leaves to the roots (Durand et al., 2016). Additionally, SWEET transporters regulate sugar supply from shoots to roots and then deliver sugars to plant growth-promoting rhizobacteria (Desrut et al., 2021). Metallothionein *MT2* gene was also upregulated and it is particularly important in stress responses, including oxidative stress and metal homeostasis (Liu et al., 2022).

In addition to transcriptome changes, the miRNAome is frequently altered under stress conditions. Our results also identified two mRNA-miRNA pairs that may be involved in biotic stress responses. In plants infected with *B.cinerea*, ath-miR168a-3p was upregulated, while its target, BDG4 (lysophospholipase BODYGUARD 4) was downregulated. Interestingly, ath-miR168a-3p was also found to be upregulated in Arabidopsis infected with another necrotrophic fungal pathogen, *Sclerotinia sclerotiorum* (Cao et al., 2020). BDG4 has been shown to be linked to cuticle-related resistance and plant defense. Arabidopsis plants with altered cuticular structures exhibited total immunity to *B. cinerea* (Bessire et al., 2007), while *bdg4* mutants displayed increased resistance to *B. cinerea* (Chassot et al., 2007; Voisin et al., 2009; Aragón et al., 2021). Beyond its role as a physical barrier, the cuticle is likely involved in signaling, and BDG4 as an integral component might play a significant role in plant-pathogen interactions, particularly against *B. cinerea*, contributing to plant resistance (Aragón et al., 2021). Our findings suggest that ath-miR168a-3p may act as a signaling event that triggers a cascade-related immune response to enhance plant resistance to *B. cinerea*.

The effect of *B. cinerea* infection in PsJN-inoculated plants is marked by downregulation of ath-miR866-3p and a subsequent upregulation of the antifungal-related gene *CRK28*, a cysteine-rich receptor-like protein kinase. In Arabidopsis, *CRK28* plays an essential role in integrating various environmental and developmental signals to regulate plant stress responses, particularly those involving abscisic acid signaling (Pelagio-Flores et al., 2019). CRK28 interacts with other components to form a pattern-recognition receptor (PRR) immune complex, which is crucial for triggering pattern-triggered immunity (PTI), including ROS production and programmed cell death. Constitutive overexpression of AtCRK28 enhanced resistance to the hemi-biotrophic bacterial pathogen *Pseudomonas syringae* pv. tomato DC3000 (Yadeta et al., 2017). In contrast, *crk28* mutants showed severe disease symptoms after infection with *Pst* DC3000 and were more susceptible to biotrophic fungal pathogens like *Golovinomyces orontii* (Zeiner et al., 2023; Zhang et al., 2023). Our results suggest that ath-miR866-3p may be involved in pathogen responses, potentially enhanced by the beneficial bacterium PsJN.

Two miRNAs in *B. cinerea*-infected plants, ath-miR158b and ath-miR858b, target transcription factors involved in responses to hypoxic stress. Ath-miR158b downregulated bHLH94, a transcription factor belonging to the bHLH family, the second largest family of plant transcription factors after MYB. It has been shown that bHLH94 was downregulated under both waterlogging and osmotic stress (Pereira-Santana et al., 2017; Nuanlaong et al., 2021). The bHLH family is particularly important in JA signaling pathways and JA-dependent responses such as flavonoid production and defense against herbivores and pathogens (Goossens et al., 2017).

Moreover, our study found that ath-miR858b targets MYB family transcription factors, which regulate various secondary metabolic pathways. MYB-like 2 and MYB20 were downregulated, while MYB13 and MYB37 were upregulated in CB compared to CNI. miR858 is commonly associated with flavonoid-specific MYB transcription factors and is involved in resistance to necrotrophic and hemibiotrophic pathogens in Arabidopsis (Sharma et al., 2016; Camargo-Ramírez et al., 2018).

Several miRNAs identified in our study have been previously linked to drought or waterlogging responses. Ath-miR157a-3p was upregulated in both non-inoculated and PsJN-inoculated plants after *B. cinerea* infection (**Supplementary Table S6**). This miRNA was also reported under drought conditions in alfalfa (Li et al., 2017). In Arabidopsis, its target gene *OHP2* (ONE-HELIX PROTEIN 2), which was downregulated in both CB and PB plants, is essential for light acclimation and photosynthesis (Beck et al., 2017).

In the PB *vs*. PNI comparison group, we found that ath-miR161.1 and ath-miR161.2 were altered, with their target genes belonging to the Pentatricopeptide Repeat (PPR) protein superfamily being both upregulated and downregulated. PPR proteins are involved in various aspects of RNA metabolism, while some PPRs play important roles in biotic and abiotic stress responses in Arabidopsis and other species (Laluk et al., 2011; Xing et al., 2018).

## 5 Concluding remarks

The capacity of PsJN in inducing ISR against *B. cinerea* was shown here on *A. thaliana* grown both in soil and in hydroponic conditions. Comparative transcriptome analysis revealed that PsJN induced differential expression of genes involved in abiotic and biotic stress responses, with the ET pathway-related genes being conspicuous in both cases. Prior to pathogen attack, PsJN modulated expression of genes involved in abiotic stress responses, microbe-plant interactions and ISR. In roots, PsJN predominantly downregulated the expression of genes related to microbe perception, signaling and immune response, indicating that PsJN locally provoked attenuation of defense responses to facilitate and support colonization and the maintenance of mutualistic relationship. In leaves, the increased expression of defense-related genes, including ET pathway genes, prior to infection, in combination with the protective effect of PsJN that is evident in later stages of infection, suggests that bacterial inoculation primes plants for enhanced systemic immune response after subsequent pathogen attack. Since it is important for the host to increase its combat readiness in advance of a disease outbreak, we hypothesize that the transcriptome changes in PsJN-treated Arabidopsis plants prior to infection enabled a more rapid response at the metabolic level when the pathogen emerged. Analyses of the proteome and post-translational modifications would provide more precise information on the role of the different defense-related genes and miRNA and their interplay in PsJN-induced resistance in *A. thaliana* against *B. cinerea*.

## Data Availability

The data discussed in this publication has been deposited in the SRA database and can be accessed through accession number PRJNA1177658 (https://www.ncbi.nlm.nih.gov/sra/PRJNA1177658).
