# Supplementary figures and images for: Paraburkholderia phytofirmans PsJN triggers local and systemic transcriptional reprogramming in Arabidopsis thaliana and increases resistance against Botrytis cinerea
Source: Front Plant Sci. 2025 Jun 3;16:1554036. doi: 10.3389/fpls.2025.1554036 (PMC12170591; doi:10.3389/fpls.2025.1554036)

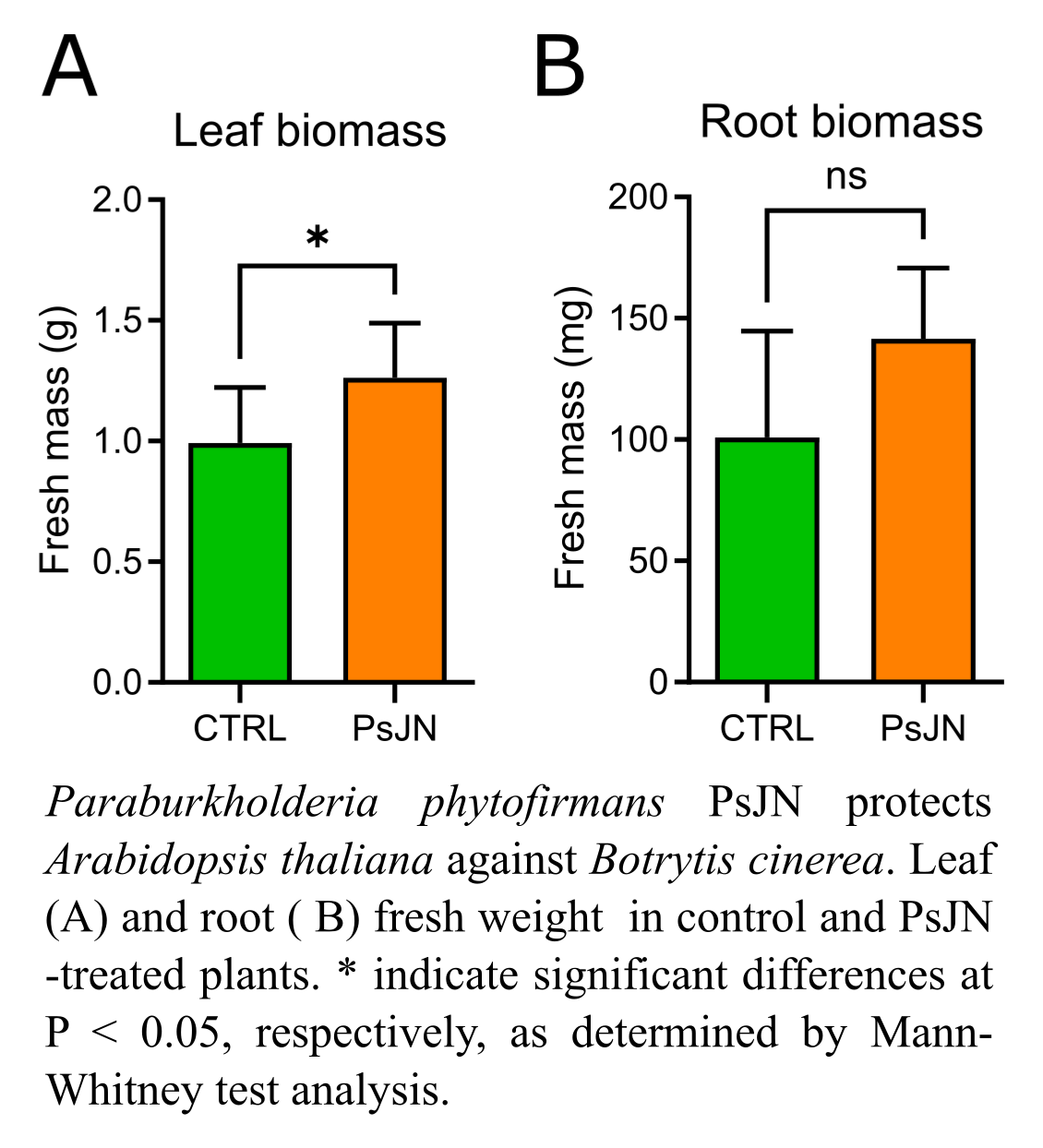

Supplement: Supplementary file 1 [file Image1.tiff]
