# Supplementary material for: Paraburkholderia phytofirmans PsJN triggers local and systemic transcriptional reprogramming in Arabidopsis thaliana and increases resistance against Botrytis cinerea
Source: Front Plant Sci. 2025 Jun 3;16:1554036. doi: 10.3389/fpls.2025.1554036 (PMC12170591; doi:10.3389/fpls.2025.1554036)
Supplement: Supplementary file 2 [file Supplementaryfile1.docx]

Supplementary Figure 1

*Paraburkholderia phytofirmans* PsJN protects *Arabidopsis thaliana* against *Botrytis cinerea*. Leaf (A) and root (B) fresh weight in control and PsJN-treated plants. * indicate significant differences at *P* < 0.05, respectively, as determined by Mann-Whitney test analysis.

Supplementary Table S1

Differentially expressed genes (DEGs) in comparisons of treatments: CNI - plants mock inoculated and mock infected, CBc - plants mock inoculated and infected with B. cinerea, PNI - plants inoculated with PsJN and mock infected, PBc - plants inoculated with PsJN and infected with *B. cinerea*.

Supplementary Table S2

MapMan analysis of the transcriptome of leaves – comparison of plants inoculated with PsJN (PNI) or mock-inoculated (CNI). Genes belonging to different pathways are sorted into different tabs: Signalling, Cell wall, Proteolysis, PR proteins, Beta glucanase and ERF transcription factors.

Supplementary Table S3

MapMan analysis of the transcriptome of roots – comparison of plants inoculated with PsJN (PNI) or mock-inoculated (CNI). Genes belonging to different pathways are sorted into different tabs: Signalling, Cell wall, Proteolysis, PR proteins, Beta glucanase and ERF transcription factors.

Supplementary Table S4

MapMan analysis of the transcriptome of leaves – comparison of *Botrytis* infected plants previously inoculated with PsJN (PBc) or mock-inoculated (CBc). Genes belonging to different pathways are sorted into different tabs: Signalling, Cell wall, Proteolysis, PR proteins, Beta glucanase and ERF transcription factors.

Supplementary Table S5

MapMan analysis of the transcriptome of roots – comparison of *Botrytis* infected plants previously inoculated with PsJN (PBc) or mock-inoculated (CBc). Genes belonging to different pathways are sorted into different tabs: Signalling, Cell wall, Proteolysis, PR proteins, Beta glucanase and ERF transcription factors.

Supplementary Table S6

Differentially expressed miRNAs in response to *P.phytofirmans* PsJN and *B.cinerea*.

Treatments: CNI - plants mock inoculated and mock infected, CBc - plants mock inoculated and infected with *B. cinerea*, PNI - plants inoculated with PsJN and mock infected, PBc - plants inoculated with PsJN and infected with *B. cinerea*.

Supplementary Table S7

Statistics of miRNA samples mapping to genome.
