# Supplementary material for: Paraburkholderia phytofirmans PsJN triggers local and systemic transcriptional reprogramming in Arabidopsis thaliana and increases resistance against Botrytis cinerea
Source: Front Plant Sci. 2025 Jun 3;16:1554036. doi: 10.3389/fpls.2025.1554036 (PMC12170591; doi:10.3389/fpls.2025.1554036)
Supplement: Supplementary file 10 [file Table8.docx]

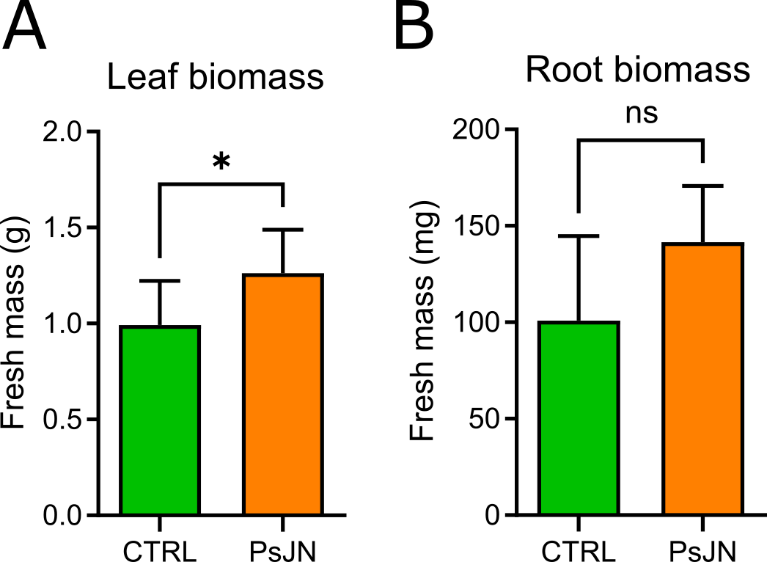


Supplementary Figure 1

*Paraburkholderia phytofirmans* PsJN protects *Arabidopsis thaliana* against *Botrytis cinerea*. Leaf (A) and root (B) fresh weight in control and PsJN-treated plants. * indicate significant differences at *P* < 0.05, respectively, as determined by Mann-Whitney test analysis.
